# Pool Boiling of Nanofluids on Biphilic Surfaces: An Experimental and Numerical Study

**DOI:** 10.3390/nano11010125

**Published:** 2021-01-07

**Authors:** Eduardo Freitas, Pedro Pontes, Ricardo Cautela, Vaibhav Bahadur, João Miranda, Ana P. C. Ribeiro, Reinaldo R. Souza, Jeferson D. Oliveira, Jacqueline B. Copetti, Rui Lima, José E. Pereira, António L. N. Moreira, Ana S. Moita

**Affiliations:** 1 IN+, Center for Innovation, Technology and Policy Research, Instituto Superior Técnico, Universidade de Lisboa, Av. Rovisco Pais, 1049-001 Lisboa, Portugal; a75320@alunos.uminho.pt (E.F.); pedrodanielpontes@outlook.pt (P.P.); ricardo.m.cautela@tecnico.ulisboa.pt (R.C.); sochapereira@tecnico.ulisboa.pt (J.E.P.); aluismoreira@tecnico.ulisboa.pt (A.L.N.M.); 2 Walker Department of Mechanical Engineering, The University of Texas at Austin, Austin, TX 78712, USA; vb@austin.utexas.edu; 3 CEFT, Faculdade de Engenharia da Universidade do Porto (FEUP), R. Dr. Roberto Frias, 4200-465 Porto, Portugal; jmiranda@fe.up.pt (J.M.); rl@dem.uminho.pt (R.L.); 4 Centro de Química Estrutural, Instituto Superior Técnico, Universidade de Lisboa. Av. Rovisco Pais, 1049-001 Lisboa, Portugal; apribeiro@tecnico.ulisboa.pt; 5 Metrics, Mechanical Engineering Department, University of Minho, Campus de Azurém, 4800-058 Guimarães, Portugal; reisartre@gmail.com; 6 Center of Technology and Innovation, University Center FSG, Os dezoito do Forte St., 2366, Caxias do Sul P.B. 65020-472, Rio Grande do Sul State, Brazil; jeferson.physics@gmail.com; 7 Mechanical Engineering Graduate Program, LETEF, Laboratory of Thermal and Fluid Dynamic Studies, University of Vale do Rio dos Sinos, Dos Sinos Av., 950, São Leopoldo P.B. 93022-750, Rio Grande do Sul State, Brazil; jaquecopetti@yahoo.com.br; 8 CINAMIL, Department of Exact Sciences and Engineering, Portuguese Military Academy, R. Gomes Freire, 203, 1169-203 Lisboa, Portugal

**Keywords:** nanofluids, pool boiling, cooling, infrared thermography

## Abstract

This study addresses the combination of customized surface modification with the use of nanofluids, to infer on its potential to enhance pool-boiling heat transfer. Hydrophilic surfaces patterned with superhydrophobic regions were developed and used as surface interfaces with different nanofluids (water with gold, silver, aluminum and alumina nanoparticles), in order to evaluate the effect of the nature and concentration of the nanoparticles in bubble dynamics and consequently in heat transfer processes. The main qualitative and quantitative analysis was based on extensive post-processing of synchronized high-speed and thermographic images. To study the nucleation of a single bubble in pool boiling condition, a numerical model was also implemented. The results show an evident benefit of using biphilic patterns with well-established distances between the superhydrophobic regions. This can be observed in the resulting plot of the dissipated heat flux for a biphilic pattern with seven superhydrophobic spots, δ = 1/d and an imposed heat flux of 2132 w/m^2^. In this case, the dissipated heat flux is almost constant (except in the instant t* ≈ 0.9 when it reaches a peak of 2400 W/m^2^), whilst when using only a single superhydrophobic spot, where the heat flux dissipation reaches the maximum shortly after the detachment of the bubble, dropping continuously until a new necking phase starts. The biphilic patterns also allow a controlled bubble coalescence, which promotes fluid convection at the hydrophilic spacing between the superhydrophobic regions, which clearly contributes to cool down the surface. This effect is noticeable in the case of employing the Ag 1 wt% nanofluid, with an imposed heat flux of 2132 W/m^2^, where the coalescence of the drops promotes a surface cooling, identified by a temperature drop of 0.7 °C in the hydrophilic areas. Those areas have an average temperature of 101.8 °C, whilst the average temperature of the superhydrophobic spots at coalescence time is of 102.9 °C. For low concentrations as the ones used in this work, the effect of the nanofluids was observed to play a minor role. This can be observed on the slight discrepancy of the heat dissipation decay that occurred in the necking stage of the bubbles for nanofluids with the same kind of nanoparticles and different concentration. For the Au 0.1 wt% nanofluid, a heat dissipation decay of 350 W/m^2^ was reported, whilst for the Au 0.5 wt% nanofluid, the same decay was only of 280 W/m^2^. The results of the numerical model concerning velocity fields indicated a sudden acceleration at the bubble detachment, as can be qualitatively analyzed in the thermographic images obtained in this work. Additionally, the temperature fields of the analyzed region present the same tendency as the experimental results.

## 1. Introduction

Pool boiling regime is a promising heat transfer phenomenon which has many applications, and needs further developments and improvements in the nanofluids field. The intrinsic capacity to transfer heat by natural convection, allied to the forced convection inducted by the detaching bubbles and the contribution of latent heat removal, makes boiling a more efficient means for heat dissipation in liquid–surface interfaces, when compared to processes with no phase change, such as pure natural convection. In addition, pool boiling systems have a simple and flexible configuration and do not require auxiliary systems, such as a pump and fan, to provide fluid flow motion, making them suitable for cooling systems for high-power dissipated loads present in the cooling of compact electronic systems and parts, propulsion in automotive vehicles, energy-conversion systems for mobility and UAVs (Unmanned Airborne Vehicles) for defense and military applications. Hence, the combination of high heat-transfer coefficients with simple configurations makes pool boiling a rising option in heat-dissipation problems. 

Nowadays, a new generation of thermofluids, known as nanofluids (NFs), are gaining increasing attention in the scientific community due to their remarkable properties at the nanoscale level. The fact that NFs exhibit higher thermal conductivity and thermal diffusivity than conventional fluids has attracted the interest of many technological fields, such as microelectronics, microfluidics, medical care, etc. [1,2]. NFs can be defined has a basefluid containing suspensions of solid nanoparticles (NPs) (<100 nm) that have significant conductivity and thermal diffusivity [3]. The basefluids more commonly used are water, ethylene glycol and oil [4]. The thermophysical properties of the nanofluids depend on the nature, size and volumetric concentration of the nanoparticles; the nature and physical properties of the base fluid; and the fabrication route, which can be a single-step method or a two-step approach [5]. The nanoparticles can be made of different materials, such as metals, ceramics and carbons suspended in a base fluid [1,2,3,4,5,6]. The size of the mixed solid particles in the base fluid is a relevant parameter to be covered in the development of nanofluids [7], which can lead to fast sedimentation and viscosity increasing. The earlier versions of colloidal fluids containing millimetric/micrometric-sized particles were unsuccessful, mainly due to the poor stability of the suspensions causing not only agglomeration and fast sedimentation but also problems related to viscosity and flow in channels with complex geometries [8]. The preparation phase regarding the ultrasonic treatment strongly influences the dispersion quality and the short-and long-term stability of the nanofluids, as well as their thermophysical properties, heat transfer and pressure drop [9]. A study revealed that parameters such as the ultrasonication time [10] can affect the aforementioned properties and also the rheological properties of the nanofluids. Moreover, predefining and monitoring the sonication bath temperature leads to the preparation of more stable nanofluids [11]. However, the introduction of NPs into the base fluids is claimed to significantly enhance the stability and heat transfer performance [1,2,3,4,5,6]. Some researchers [12,13] have observed a great increase of the NFs’ thermal conductivity, when compared to conventional coolants; moreover, an enhancement of the convection, which can be natural, forced or mixed [14], in the nanofluids was also reported by References [15,16], in which grew interest for industrial applications. For example, due to physical space, weight and energy constraints available in space stations and aircrafts, there is an increasing scientific interest to develop the smallest and most efficient heating/cooling system [17].The NFs with very high heat fluxes that can provide the necessary heating/cooling rates have opened strong possibilities for simplifying cooling requirements for space industry applications [18,19]. Another example is the magnetic nanoparticles (MNPs) applied in magnetic fluid hyperthermia. This technique is considered to be promising among the therapeutic techniques for the treatment of cancer, as it implements the remarkable nanoscale physicochemicalproperties of MNPs that can generate heat under an alternating magnetic field [20,21]. 

However, it is widely accepted by the scientific community that the NFs’ long-term stability is one of the major factors that is slowing down the industrial applications of the NFs [2,4]. In addition, several other parameters influence the thermal performance of NFs and need to be investigated.

In order to improve pool boiling efficiency, some induced surface modifications were tested in the last years. Some properties, such as the surface wettability, have shown the ability to improve pool-boiling heat transfer by increasing the heat-transfer capacity at low superheat values. As wettability affects the dynamic of the bubble formation process, it is considered to be an essential factor in pool boiling studies [22,23]. In this context, several authors have focused on the customization of surface properties to improve pool-boiling heat-transfer processes. In a recent approach, the so-called biphilic surfaces, i.e., hydrophilic/superhydrophilic surfaces with hydrophobic/superhydrophobic regions or spots, show great potential to enhance heat transfer coefficients (HTC) and delay the occurrence of the critical heat flux (CHF). 

Recently, the work reported by Reference [24] made it possible to develop superbiphilic surfaces and to report an increase in the CHF and enhanced heat transfer. The authors of that work investigated the pool-boiling heat transfer by using aluminum superbiphilic surfaces for saturated water at atmospheric pressure. The triangular lattice pattern of superhydrophobic circular spots was used with spot diameters ranging between 0.25 and 1.0 mm and pitch values from 0.5 to 2.5 mm. The best heat-transfer performance is achieved by using 0.5 mm diameter spots, a spot pitch of 1 mm and a corresponding superhydrophobic area fraction of approximately 23%. The work reported by Reference [25] postulated that, if the average bubble departure diameter can be reduced, both CHF and HTC can be enhanced, owing to the reduced dry-spot area and increased active bubble cycle. Adjusting the hydrophobic pattern size and the pitch of the biphilic surface by using a porous superhydrophobic material with high adhesion to vapor, the authors obtained an enhancement of 14.5% and 34.1% for the CHF and HTC, respectively, using a S2P4N64 biphilic surface, when compared to the bare surface. In the study of Reference [26], the researchers showed how the biphilic surface can regulate the liquid and vapor transport from the heating surface and be responsible to increase the heat transfer and delay critical heat flux. According to the findings of Reference [23], this can be achieved by juxtaposing the hydrophilic and hydrophobic regions for the biphilic surface.

Apart from a few exceptions focusing on the interpretation of the obtained boiling curves, based on bubble dynamics, combining high-speed visualization and thermography [27], most of these studies address a trial-and-error approach to create a biphilic pattern, which is tested to infer on if it leads to higher heat transfer coefficients. An accurate description of the intricate relations between bubble dynamics in basic patterns and the associated heat transfer processes occurring during bubble growth and departure are now considered to be vital to devise complex surfaces [28]. 

An analysis of bubble dynamics was performed by Reference [29] on different samples, through high-speed imaging, using three intervals of heat flux. The researchers evaluated the effect over the heat transfer, comparing a homogeneous plain copper surface with a biphilic surface with hydrophobic patterns. An enhancement up to 1.16 was reached by the biphilic surface, whereas that with superhydrophobic patterns decreased to 0.83 at the highest evaluated heat flux. The authors explain that the surface wettability led to different bubble dynamics, which should be responsible for the difference in the results. The physical mechanism for the ultimate takeover of intermittent boiling on heterogeneous wettability-patterned surfaces of low-pressure biphilic surfaces, using pure water, was investigated by Reference [30]. As a result, the authors reported that the three-phase contact line was more likely to be dislodged from the edge of the hydrophobic spot under sufficiently low-pressure conditions, where particularly quick bubble growth tended to prevail. An investigation to find a novel and facile process flow for the fabrication of biphilic surfaces was proposed and tested by Reference [31]. The researchers studied ten biphilic surfaces with hydrophilic/total areas ranging from 0.19% to 95% and they tested them, to analyze the effect of heterogeneous wettability. According to the results, the heat transfer coefficient and critical heat flux increased with the increasing of hydrophilic/total areas up to 38.46%. On the other hand, surfaces with hydrophilic/total areas greater than 38.46% demonstrated a decreasing trend in CHF and heat transfer coefficient enhancement, which was caused by the earlier interaction of nucleated bubbles.

The current work aimed to investigate the combination of customized surface modification with the use of nanofluids, in order to enhance the heat transfer on pool boiling regimes. As the objective, it can be stated that this study intended to develop a customized biphilic surface composed of hydrophilic surfaces patterned with evenly separated superhydrophobic spots. Another objective was to use that biphilic surface to act as a surface interface with different nanofluids (water with gold, silver, aluminum and alumina nanoparticles) and evaluate the effect of the nature and concentration of the nanoparticles in bubble dynamics and hence in the pool-boiling heat-transfer process. The analysis performed combines high-speed visualization with infrared thermography, to derive a detailed description of the bubble dynamics phenomenon, which is then used to explain the obtained surface temperature fields and local heat-transfer processes. In addition, a numerical model for nucleate boiling, using biphilic surfaces, was implemented to be compared with the experimental results. To the best of our knowledge, there is a considerable research gap in the state-of-the-art in the field related to the present investigation, especially with the understanding of the thermal behavior of nanofluids on biphilic surfaces, under pool boiling scenarios. 

## 2. Materials and Methods

### 2.1. Nanofluid Preparation

Nanofluids were prepared as solutions of distilled water with nanoparticles of alumina, aluminum, silver and gold, with mass concentrations varying between 0.05 and 1 wt%. The gold and silver nanofluids are transparent and prepared by using a one-step method. After weeks of preparation, the solutions did not deposit particles at the bottom of recipients, thus revealing good stability. The aluminum and alumina nanofluids were prepared by using a two-step method. They are opaque, which impedes from acquiring data with the high-speed camera and also hinders the data acquisition with the IR camera, since it is not possible to synchronize the data with dynamic images. To solve the sedimentation problem, it was found that, through a probe-type ultrasonication, it was sufficient to keep the fluids stable for more than one day. Thus, we used a probe-type ultrasonic homogenizer (Model *UP200Ht* from *Hielsher*) with a frequency of 25 KHz and a power of 100 W, for a sonication process with a duration time of 90 minutes.

All the solutions were characterized in terms of density, viscosity, specific heat, conductivity and surface tension. These properties were evaluated at room temperature (20 °C ± 3 °C), except for conductivity, which was measured at different ambient temperatures, between 20 and 60 °C. Table 1 shows the thermophysical properties for the different working fluids used in this study. The values depicted in Table 1 were obtained by applying the following formulations: (a) the density (ρ) is calculated by using the correlation presented by Sezer et al. [32]; (b) the specific heat capacity ($C_{p}$) of the nanofluids is given by Xuanand and Roetzel [33]; and the surface tension (σ) was determined by the pendant drop method, using the optical tensiometer THETA (*Attension*), with the result being an average value of 15 measurements from each fluid. 

The properties depicted in Table 1 and other thermophysical properties, such as the viscosity and the thermal conductivity, can define relevant parameters present on pool boiling regimes, like the boiling heat transfer and the critical heat flux for nucleate pool boiling [34]. Except for the thermal conductivity and for the specific heat, all the other properties were evaluated experimentally in previous works [35,36], to validate the theoretical approaches used. The thermal conductivity was estimated by using the Maxwell model [37]. Since the volume fraction of the nanoparticles in the base fluid was very small, the obtained values were almost identical to the thermal conductivity of water, so it is not included in Table 1. It is worth mentioning that the results are mainly explored by taking into account the boiling phenomena and convective motion, so that thermal conductivity plays a minor role in the analysis. The modifications are shown in the manuscript in blue.

It is worth mentioning that the Maxwell model [37] was used to obtain the thermal conductivity:

(1) $$K_{nf_{Maxwell}}=K_{f}\left(\frac{K_{np}+2K_{bf}+2\varphi \left(K_{np}-K_{bf}\right)}{K_{np}+2K_{bf}-2\varphi \left(K_{np}-K_{bf}\right)}\right)$$

In this equation$,\;K_{np}$, $K_{bf}$ and $K_{nf_{Maxwell}}$ are nanoparticles’ thermal conductivity, base fluid thermal conductivity and nanofluids’ thermal conductivity by the Maxwell model; φ corresponds to nanoparticles concentrations. It is possible to verify through Equation (1) that, for concentrations of up to 1% of nanoparticles, the term in parentheses in Equation (1) is approximately equal to 1, making the conductivity of the base fluid dominant. Only at concentrations greater than 1% does the thermal conductivity of the nanoparticles begin to have greater influence. Even for other models, the trend would be repeated. On the other hand, previous authors [38,39] have studied the thermal conductivity enhancement of alumina in water and demonstrated a nonlinear relationship with respect to temperature, volume fraction and nanoparticle size. The most significant finding was the effect that variations in particle size had on the effective thermal conductivity of the Al_2_O_3_/distilled water nanofluids, because it was not remarkable, as it has been reported for MWCNT. 

The stability of metallic nanoparticles based nanofluids, like those prepared in this work, can be predicted by its correlation with time-dependent factors, such as the evaporation. In the study of Reference [40], the surface tension of that kind of nanofluids was investigated in time and revealed that it decreases during evaporation. Consequently, the concentration of nanoparticles increased during the same time, and the nanofluids became less stable. In this work, mostly stability was visually observed.

### 2.2. Biphilic Surfaces Preparation 

The biphilic surfaces were prepared on a 20 µm thick stainless-steel foil (AISI304) and with well-defined rectangles of the dimensions 50 × 38 mm^2^. The biphilic patterns were obtained by applying a mask on the foil and then spraying with a superhydrophobic coating. The diameter of the superhydrophobic regions was 1.5 mm, while the distance between them was fixed to be the size of the bubble characteristic diameter. Two electrical wires are welded to the contacts and fixed to the female connectors, which were isolated and set apart from the base by means of ceramic washers. Following this, two layers of high-temperature-resistant black spray were deposited on the surface, through the hole at the bottom of the base. The layers were made within a 15 min interval and allowed to dry for 2 h, in air, at room temperature. To be used as a stencil, two different options were followed. The first evolved a 3D-printed part with 19 holes of 1.5 mm in diameter and evenly separated between them by a 1.5 mm spacing. The second option was using the same configuration on a steel sheet, where the holes are done by laser. In spite of being thinner, which is better to stop the formation of deposit while spraying the surface, the first option produces better results. This is explained by the fact that the sheet is difficult to stretch and therefore some superhydrophobic spray flows beneath its holes. The distance between holes was selected to be of the order of one bubble diameter. Although in this work only single and septuple spots are tested, the configuration with 19 holes raises the possibilities of tests that one can conduct. After having the stencil printed, 6 layers of NeverWet spray were applied to the surface and allowed to dry and cure for 12 h, in air, at room temperature. The final step consisted of placing two layers of Kapton insulating tape. After this, all the excess spray and any other impurities on the biphilic surface were cleaned with acetone. The final result was the base of the tank ready to be attached to the rest of the setup.

### 2.3. Surface and Fluid Characterization 

The biphilic surfaces and working fluid used in this study were characterized in terms of wettability and surface tension. The wettability is defined by the measurement of the following parameters: static contact angle ($\theta_{s}$), quasi-static advancing angle ($\theta_{a}$), quasi-static receding angle ($\theta_{r}$) and contact angle hysteresis $\left(∆\theta \right)$. The equipment used to measure those parameters was the optical tensiometer THETA (*Attension*), and the followed method was the sessile drop one, as described in previous published works of the researching group [41,42]. All the mentioned parameters were measured at room temperature (20 °C) and repeated, to ensure that the coat was evenly distributed. Table 2 summarizes the obtained mean values and respective standard deviation (SD). 

Given the fact that the contact angle is dependent of the surface tension between the different existing phases in pool boiling (solid, liquid and vapor), it was important to determine the surface tension of all the fluids in presence. For this purpose, once again, we used the optical tensiometer THETA. The methodology is essentially the same as the one used for measuring the contact angle, except for the fact that, this time, a pendant drop was analyzed. The obtained values are depicted in Table 3. The values presented in Table 3 resulted from the average value of 15 measurements for each fluid.

### 2.4. Experimental Setup

**Figure 1:**
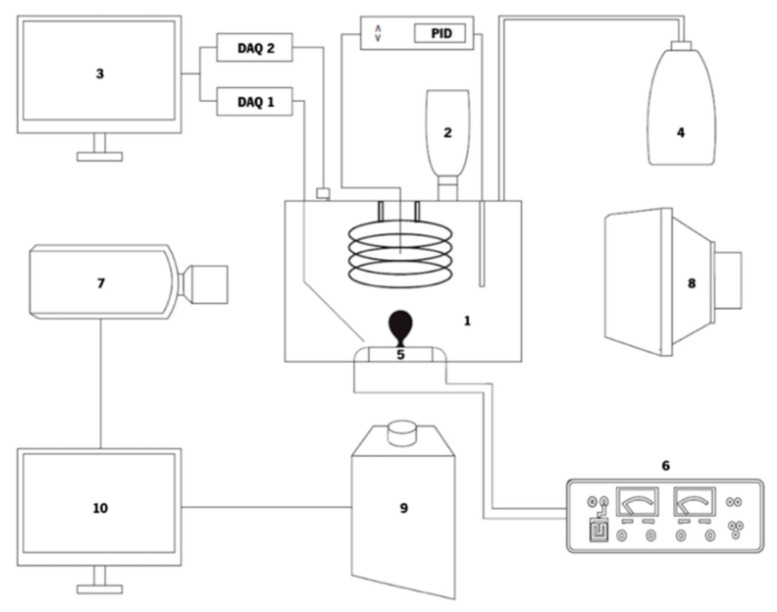
Schematic view of the experimental setup: (**1**) boiling chamber, (**2**) funnel, (**3**) PC, (**4**) condensed fluid recipient, (**5**) tank base, (**6**) DC power supply, (**7**) high-speed camera, (**8**) LED backlight, (**9**) IR camera and (**10**) PC.

**Figure 2:**
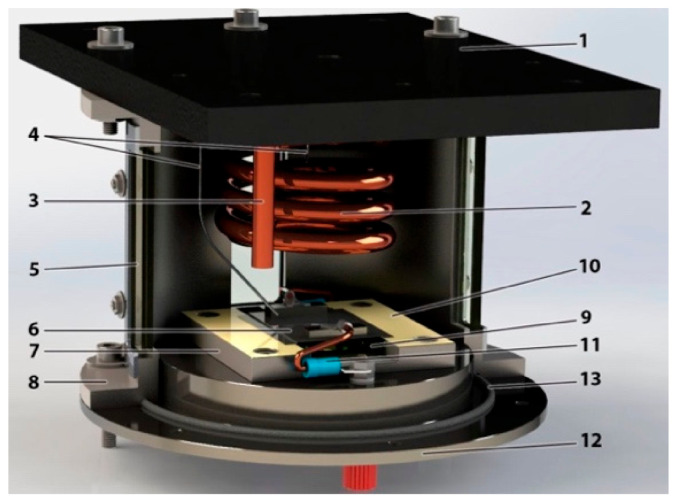
Assembled model of the boiling chamber and its components: (**1**) support, (**2**) coil heater, (**3**) cartridge heater, (**4**) thermocouples, (**5**) window, (**6**) test surface, (**7**) test surface base, (**8**) tank, (**9**) thermal glass, (**10**) Kapton tape, (**11**) copper wire, (**12**) tank base and (**13**) O-ring.

The experimental setup is schematically illustrated in Figure 1. The schematic view of Figure 1 shows the boiling tank (item 1), where the experiment is performed and observed. The fluid is heated using two resistance heaters—a coil and a cartridge heater. The tank is filled by using a funnel (item 2) connected to a feeding valve. The temperature is measured by two types of K thermocouples. To control the temperature of the fluid inside the boiling chamber, a proportional-integral-derivative controller (PID) is connected to the cartridge heater and to a thermocouple, shutting down the resistance heater when it gets to a defined value. One pressure sensor and one thermocouple are connected to the data-acquisition equipment (DAQ), which sends the information to a PC (item 3). An open system keeps the water vapor flowing through a tube, to a recipient, where its condensates, maintaining the boiling chamber at atmospheric pressure (item 4). More details of the boiling chamber can be seen in Figure 2.

The test surface, an AISI304 stainless steel foil, was located at the base of the boiling chamber (item 5) and it was the place where the nucleation phenomenon occurred. The stainless steel foil is heated by means of Joule effect, by applying current directly to its surface, using a HP6274B DC power supply (item 6). The values of the current, which varied between 3A and 9A, imposed a heat flux to the heating surface ranging between 0.025 and 0.229 W/cm^2^.

Bubble dynamics and heat transfer were characterized from post-processing of synchronized high-speed and thermal images, using a high-speed camera (Phantom v4.2) (item 7), placed on a frontal glass window of the boiling chamber, with an LED backlight (item 8) that illuminates the boiling chamber. An infrared camera (Onca MWIR-InSb-320) (item 9), was placed below the surface. The frame rate of the high-speed camera was set to 2200 fps, while the high-speed infrared camera images were recorded at 1000 fps. The selected pixel size for the optical arrangements was of 100 µm for the infrared camera and of 40 µm for the high-speed camera. The cameras are synchronized and are both connected to a PC (item 10) where the images taken from the live analysis are collected and saved for further interpretation.

### 2.5. Numerical Method

#### 2.5.1. Numerical Model

The numerical model was covered with the assistance of the software for Computational Fluid Dynamics (CFD) *OpenFOAM*. By being an open-source software, it enables users to modify and program the different C++ language libraries and thus create new solvers. In this case, the solver *interThermalPhaseChangeFoam* [43] was selected because it is suitable for the two incompressible and non-isothermal immiscible fluids that it were in presence.

The governing equations of the problem are the mathematical formulation of the conservation laws of physics, for an infinitesimal element of fluid volume, which comprises the principle of mass conservation, the Newton’s second law of motion and the first law of thermodynamics [44]. 

So, for mass conservation the solver assumes uniform density in each phase and adds a source term to represent the dilatation rate. The continuity equation is given by the following:

(2) $$\nabla \cdot \vec{U}={\dot{\nu }}_{pc}$$

where $\vec{U}\;$ represents the velocity vector and ${\dot{\nu }}_{pc}$ is the volume source rate per unit volume.

Newton’s second law states that the variation of momentum of an element is equal to the sum of the forces being applied to it. Regarding fluid flow, these changes in momentum occurs from pressure forces, viscous forces and gravity force. For Newtonian fluids, and assuming incompressible flow, one gets the following equation for momentum conservation:

(3) $$\frac{\partial (\rho \vec{U})}{\partial t}+\nabla \cdot (\rho \vec{U}\vec{U})=-\nabla p^{\prime }+\nabla \cdot [\mu (\nabla \vec{U}+{\nabla \vec{U}}^{T})]+\rho \vec{g}+{\vec{f}}_{v}$$

where $p^{\prime }$ is the corrected pressure for hydrostatic variations, $\vec{g}$ is the acceleration of gravity and ${\vec{f}}_{\sigma }$ the volumetric surface tension force in the vicinity of the interface.

**Figure 3:**
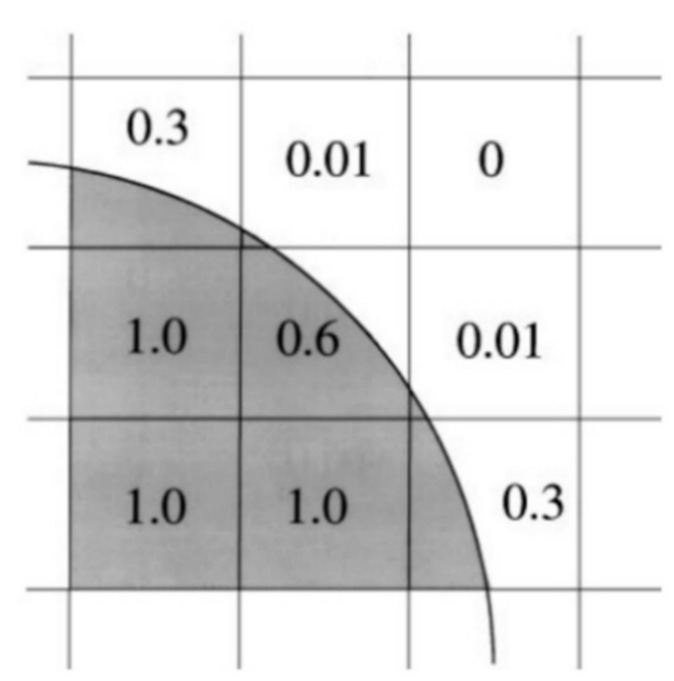
Interface representation in the VOF method [39].

Since this is a multiphase problem (two phases), a model is needed to track the interface. The solver uses the Volume-of-Fluid (VOF) method, which is widely established and used for applications where the position of the interface between the phases is of importance. The main idea is to outline a function that defines the volume fraction of a fluid in each cell [45]. This function takes the value of 1 if the cell is filled with fluid, 0 if it is filled with vapor and a value between 0 and 1 at the interface, as illustrated in Figure 3.

The surface tension is calculated by using the continuum surface tension model developed by Brackbill [46]:

(4) $${\vec{f}}_{\sigma }=\sigma k\nabla \alpha$$

Being σ the surface tension, k the local mean interface curvature and α the volume fraction. The latter takes the value 0 for the vapor phase, between zero and one for the interface and one for the liquid phase.

The local mean interface curvature can be defined by the following:

(5) $$K=-\nabla \cdot \vec{n}=\;-\nabla \cdot \frac{\nabla \alpha }{│\nabla \alpha │}$$

where $\vec{n}$ represents the vector normal to the interface.

The first law of thermodynamics states that the energy variation of a fluid element is equal to the sum of the net of the heat exchanged and the net of work exerted on that element. Considering this, the following internal energy equation can be defined as follows:

(6) $$\frac{\partial \left(\rho i\right)}{\partial t}+\nabla \cdot (\rho i\vec{U})=\nabla \cdot \left(k_{eff}\nabla T\right)-{\dot{q}}_{pc}$$

where i  represents the thermal energy, $k_{eff}$ the effective heat transfer coefficient and ${\dot{q}}_{pc}$ the phase change heating rate.

The equation for the volume fraction is now added to the three main ones defined previously and related with continuity, momentum and energy. This equation gives the volume fraction (α) at each cell and is defined by the following:

(7) $$\frac{\partial \alpha }{\partial t}+\nabla \cdot (\alpha \vec{U})-\nabla \cdot [\alpha \left(1-\alpha \right){\vec{U}}_{r}]={\dot{\alpha }}_{pc}$$

Being ${\dot{\alpha }}_{pc}$ the term of the phase generation due to phase change and ${\vec{U}}_{r}$ an artificial compression velocity. The latter term is added to count with the sharpening of the interface between the fluids, which is vital for two-phase flow of immiscible fluids problem solving [47] and is given by the following:

(8) $${\vec{U}}_{r}=min\{C_{a}|\vec{U}|,\;\max(\left|\vec{U}\right|)\}\vec{n}$$

where $C_{a}$ is the compressibility factor.

The fluid properties μ, ρ and $k_{eff}$ are calculated as arithmetic phase-fraction weighted averages of the two phases at each mesh cell and given by the following equations:

(9) $$\mu =\alpha \mu_{1}+\left(1-\alpha \right)\mu_{v}$$

(10) $$\;\;\rho =\alpha \rho_{1}+\left(1-\alpha \right)\rho_{v}$$

(11) $$\;k_{eff}=\alpha k_{1}+\left(1-\alpha \right)k_{v}$$

Finally, the terms ${\dot{\nu }}_{pc}$ and ${\dot{\alpha }}_{pc}$ are given by the following equations:

(12) $${\dot{\nu }}_{pc}=\frac{{\dot{q}}_{pc}}{i_{lv}}\left(\frac{1}{\rho_{v}}-\frac{1}{\rho_{l}}\right)$$

(13) $${\dot{\alpha }}_{pc}=-\frac{{\dot{q}}_{pc}}{\rho i_{lv}}$$

where $i_{lv}$ is the enthalpy of phase change.

The phase change heating rate (${\dot{q}}_{pc}$) calculation is done by the Interface Equilibrium Split Dilation model, which is based on the work of Rattner [48]. 

#### 2.5.2. Geometry and Mesh

The bubble growth and detachment process in a biphilic surface can be considered to beaxisymmetric. For this reason, an axisymmetric computational domain is created, with a wedge-type geometry representing a section of 5° of the entire 3D domain. This allows to simplify the geometry, while also making the simulation much less computational endeavoring. 

**Figure 4:**
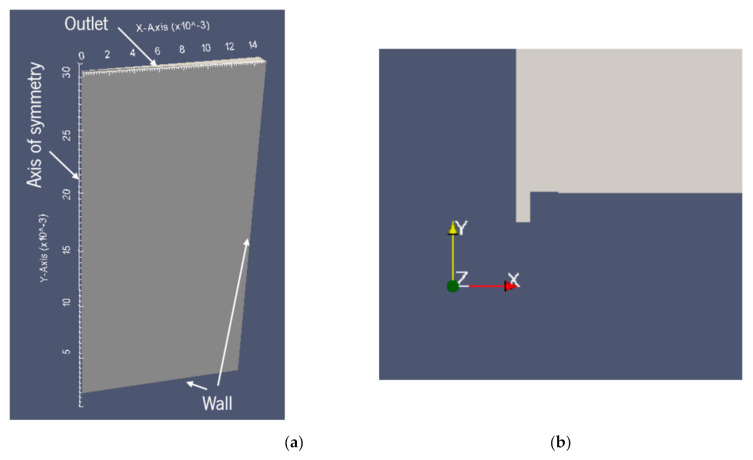
Geometry definition of the computational domain. (**a**) General geometry definition. (**b**) Nucleation cavity detail.

The dimensions of the domain are an important part of the problem definition. In order to define them, preliminary simulations with different computational domain sizes were run. Since the goal is to obtain faster results with the maximum accuracy, the domain has the minimum size required for a bubble to grow free of the boundary effects influence. Although it was possible to get bubble detachment and a somewhat similar phenomenon to the experimental results, the bubble growth is very unstable. A certain degree of instability is expected since there are always convection currents occurring in ever pool boiling scenario. However, the instability observed in preliminary simulations, especially at the boundary between the superhydrophobic and hydrophilic surfaces, was too significant to be ignored and can be attributed to an improper definition of the domain size. For this reason, the domain size in the XY plane had 15 × 30 mm^2^ of dimensions to avoid the effect of the computational domain on the result. Since the superhydrophobic region has a diameter of 1.5 mm and the bubble at detachment has a diameter of approximately 3 mm, a height of 10 times the final diameter and a width of 5 times the final diameter were chosen. These values were based on the preliminary simulations and were consistent with some bubble rise studies that can be founded in Reference [38]. To finalize the geometry definition, it is important to note that a small cavity is added on the nucleation spot next to the axis. The cavity is used to initialize the vapor phase, which is needed for this solver. The cavity size in the XY plane is 0.1 × 0.2 mm^2^. The geometry definition and the cavity are depicted in Figure 4a,b, respectively.

In order to solve the governing equations using the finite volume method, the domain must be discretized into a mesh of cells. This mesh is fundamental to ensure a robust solution for the numerical model. 

**Figure 5:**
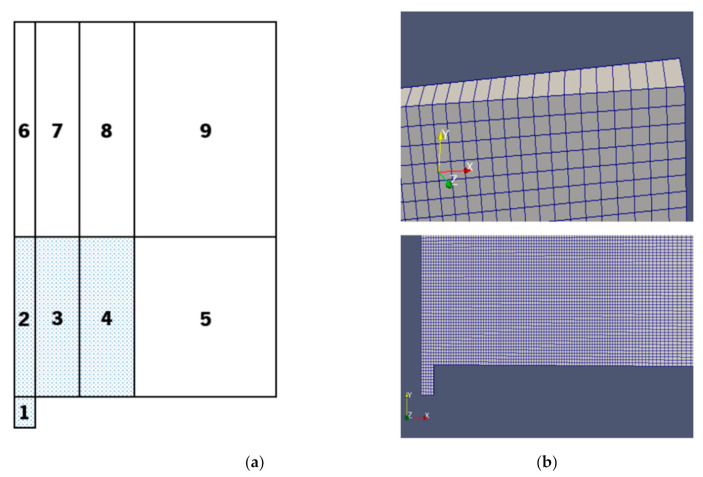
Mesh definition. (**a**) Different mesh zones of the domain. (**b**) Mesh details.

To guarantee mesh-independence, while minimizing simulation time, two different mesh zones are defined. The zone where the bubble grows is meshed with a uniform cell size, which means there is no grading between the cells. This ensures a quality mesh in the region of interest, which is particularly important in the VOF method in order to have a well-defined interface between the two phases. The second zone has a grading of 20, which means the cells in the top and right boundary are 20 times larger than the ones in the bubble growth region. This reduces the computational effort of the simulation, while preserving a smooth transition between the cells. The first zone is represented in blue and the second one in white, in Figure 5a.

For the mesh to be constructed using blockMesh, it is necessary to subdivide the mesh in 9 different zones. This is because blockMesh needs rectangular zones that are congruent with each other in order to generate the mesh. The 9 different zones are presented in Figure 5a. It should be noted, however, that not all the zones were reproduced accordingly with the right scale, for the sake of visibility. 

The final result is a non-uniform structured mesh with local refinement, consisting of 79,402 cells. All of them are hexahedral, except the 446 prisms at the axis. The minimum cell size is of 20 µm, and the maximum one (at the top right corner) is of 400 µm. Some mesh details are depicted in Figure 5b. 

#### 2.5.3. Transport Properties, Initial and Boundary Conditions

The transport properties of the liquid phase are already defined in Section 2.1, by Table 1. It is now necessary to define the vapor properties. These vapor properties are depicted in Table 4. It should be emphasized that the acceleration of gravity is considered and the simulation is of laminar type. 

**Figure 6:**
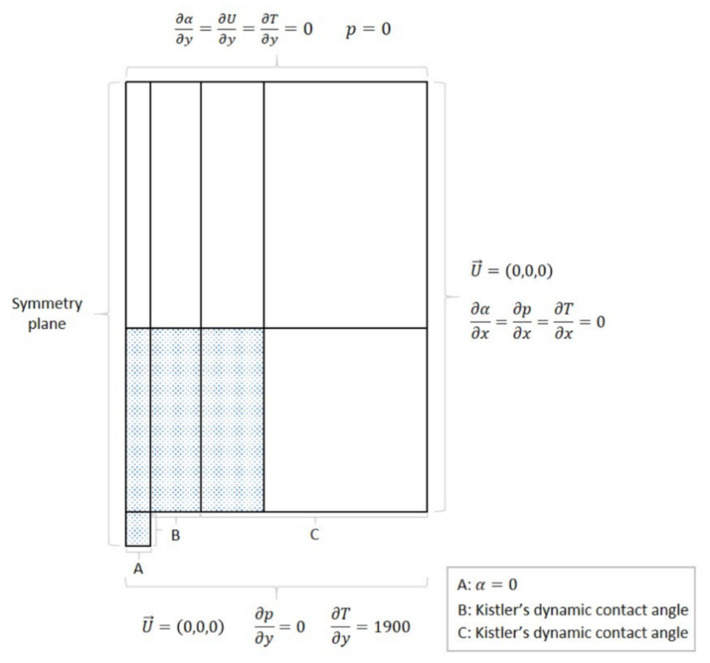
Boundary conditions of the problem.

The imposed initial conditions must represent the conditions in the tank at the start of the pool boiling phenomena. Therefore, the pressure (*p*) is defined in the domain as atmospheric pressure, the velocity (*u*) is considered zero and the domain is all at saturation temperature (*T*). For the initial conditions of the volume fraction (*α*), the function *setFields* is used. This allows to make α equal to 1 (liquid) in all of the domain except for the nucleation cavity, where the vapor phase (*α* = 0) is imposed. Thus, the boundary conditions are defined in order to complete the setting of the problem. The locations where it is necessary to define boundary conditions are depicted in Figure 6. It is important to note that this scheme is not reproduced with the right scale. 

The wall represented by letter A depicts the cavity where the nucleation is initialized. For this reason, the volume fraction (α) is defined with a *fixedValue* boundary condition with a value equal to 0 (vapor). The pressure (*p*) is defined with a *fixedFluxPressure* boundary condition and the velocity (*u*) with a *noSlip* one. The temperature (*T*) is defined with a *fixedGradient* boundary condition in order to account for the constant heat flux imposed on the surface by Joule effect. The gradient value is obtained by dividing the heat flux values (q”) by the thermal conductivity of the fluid. For a heat flux of 1290 W/m^2^, the temperature gradient value is 1900. 

The boundary conditions of the wall represented by letter B define where the bubble interface grows until starting to raise vertically—the superhydrophobic region. Hence, the volume fraction will change as the bubble grows towards the hydrophilic region. The boundary conditions for temperature, pressure and velocity are the same as the ones for the cavity. However, the volume fraction is different. Since we have a superhydrophobic coat, a contact angle value is necessary to be defined. The solver uses Kistler’s dynamic contact angle model [49], hence a *dynamicKistlerAlphaContactAngle* boundary condition is defined. Having in mind the values measured in Table 2 (see in Section 2.3), the advancing angle value is defined at 160° and the receding angle at 158°.

The wall illustrated by letter C represents the hydrophilic part of the surface. Therefore, the conditions are the same as the ones used for B but with an advancing contact angle value of 85° and a receding contact angle of 34°.

The wall located on the right part of the geometry defines the outside wall of the tank. Thus, the boundary conditions are very straightforward. For the volume fraction, temperature and pressure, a *zeroGradient* boundary condition is defined. This means that the properties of the elements at the wall have the same value as the element just before them. For the velocity, a *noSlip* boundary condition is applied. 

Since the system is open to the atmosphere, the line at the top of the scheme represents an outlet patch. Therefore, an *inletOutlet* boundary condition is defined for the volume fraction and the temperature. This type of boundary condition prevents the occurrence of any backflow at the outlet. For the same reason, a *totalPressure* boundary condition is imposed for the pressure. The velocity has a *zeroGradient* boundary condition. 

Finally, the left wall represents the axis of the geometry. Hence, a *symmetryPlane* boundary condition is defined for all the properties. 

The Supplementary Materials presents the sources for the uncertainties related to the bubble diameter measurement, the values of the uncertainties regarding with the parameters of the bubble dynamics characterization and the values of the uncertainties associated with the measurement with different types of equipment.

## 3. Results and Discussion

**Figure 7:**
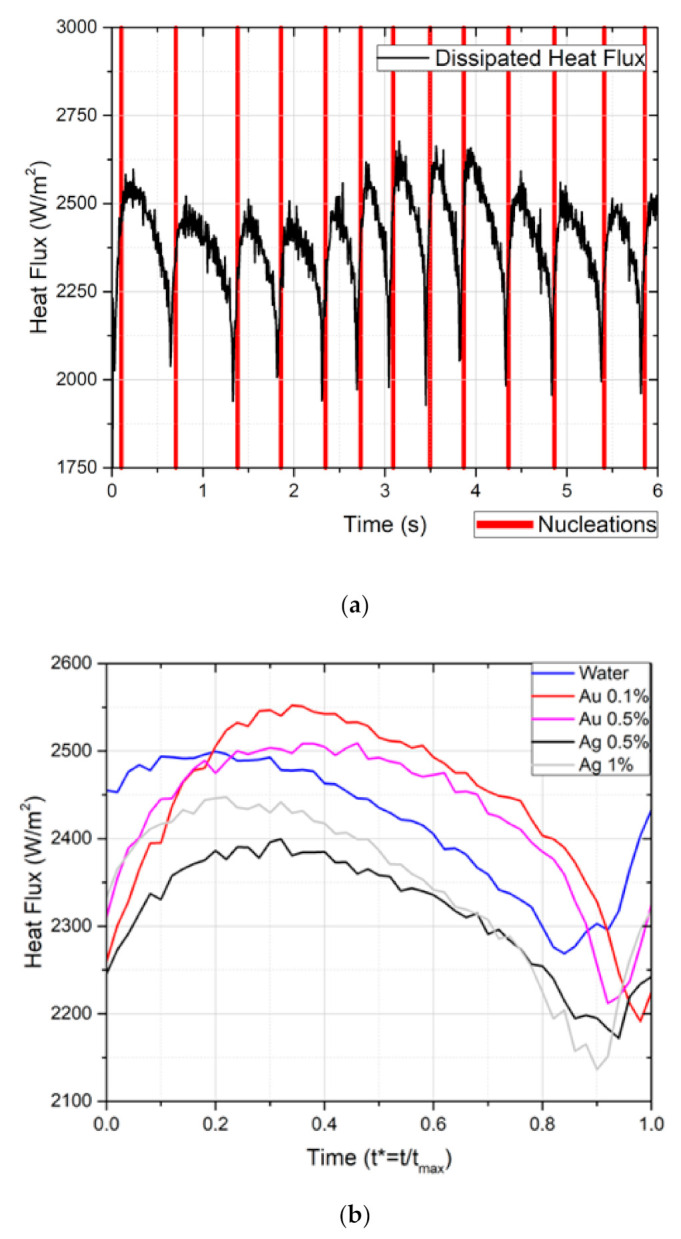
(**a**) Measured dissipated heat flux during pool boiling for a single bubble analysis of Ag 1 wt%, synchronized with the bubble departure events (red vertical lines). The imposed heat flux is 2132 W/m^2^. (**b**) Temporal evolution of the heat flux averaged from all the bubble events characterized in (**a**).

Images obtained by high-speed visualization and time-resolved thermography are consistent with those recently reported by Reference [50]. Hence, there is a peak in the heat flux that is dissipated at each event of bubble departure, as a result of the induced fluid convection. This is evident in Figure 7a,b, which represents the measured dissipated heat flux synchronized with the bubble departure events and the heat flux averaged from all of these events, for the various nanofluids tested here, respectively. The dissipated heat flux is always higher than the flux imposed on the surface because only the superhydrophobic region and the neighboring hydrophilic region are analyzed. Since this area promotes heat dissipation, the local heat dissipation is enhanced.

The dissipated heat flux seems to increase at the beginning of the nucleation, reaching a maximum value between t* *= t/t_max_* ≈ 0.2 and t* ≈ 0.4 (*t_max_* represents bubble detachment instances observed in the high-speed images). The heat flux tends to increase from the necking phase (t* ≈ 0.95) until the start of the vertical elongation (t* ≈ 0.4) of the bubble, which is in agreement with the detailed description of bubble dynamics reported in Reference [48]. After that, the dissipated flux decreases. As the bubble grows, the phase change is less prominent due to the local vapor saturation, hence the observed flux decreases. Finally, an inflexion point is reached at t* ≈ 0.95 and the heat dissipation begins to increase again. This timeframe corresponds to the necking stage. 

The strangulation of the bubble near the base occurs due to the surface tension and seems to promote heat transfer, decreasing the vapor mass directly on the top of the surface. This promotes the recirculation of the fluid and hence the increasing of the flux on the detachment near region. These earlier results suggest a minor effect of the nanofluids, which can be explained by the low concentration used at this stage of the work (to assure the preparation of a stable nanofluid). When the study reported by Reference [51] experimentally investigated the periodic growth of a single bubble and the outlet of a small heater submerged in a nanofluid containing moderately hydrophilic nanoparticles (for nanofluids made of moderately hydrophilic silica nanoparticles dispersed in deionized water), similar results were obtained. Some authors shown that the bubble departure frequency and boiling heat transfer by latent heat were enhanced by increasing concentration, but the periodic single bubble departure diameter is not affected by the presence of moderately quantities of nanoparticles in the nanofluids.

**Figure 8:**
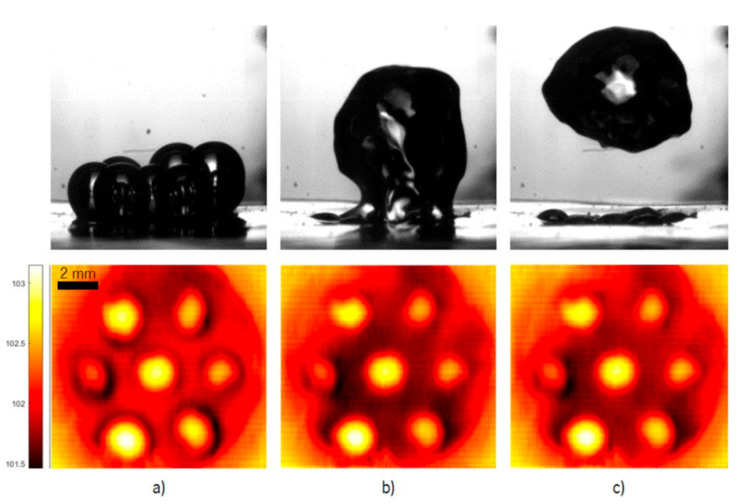
Synchronized images of the dynamic evolution between seven bubbles with spacing *δ* = 1*d* and the respective thermographical images. The imposed heat flux is 2132 W/m^2^, and the fluid is Ag 1%. (**a**–**c**) Conditions before, during and after coalescence, respectively.

On the other hand, these results suggest the enhancement of fluid motion near the hydrophilic region of the heated surface, promoted by bubble detachment, which may be enhanced using a pattern of superhydrophobic regions as discussed in the following sub-paragraphs. The results discussed in this case of a surface patterned with multiple superhydrophobic regions were obtained for the nanofluid with 1%wt Ag. The synchronized high-speed and thermographic images shown in Figure 8 evidence a clear coalescence between the bubbles. 

The images in Figure 8 illustrate the evolution of the dynamic of the bubbles and the respective thermographical images. The distance between the bubbles is *δ* = 1d and the maximum imposed heat flux is (2132 W/m^2^). The timeframe between the images is very small (60 ms) and corresponds to the period between the beginning of the necking phase and the bubble detachment. The coalescence occurs when the individual bubbles are in the necking phase and reaching the maximum diameter. The effect of the interaction of the bubbles is also noticeable in the thermographical images. 

In the first subfigure, the bubble at the superhydrophobic regions are at the beginning of the necking phase, and it is already very clear the effect that the existing multiple superhydrophobic regions have on the biphilic surface. This is caused by the multiple nucleation spots, which enhance the heat transfer and control the superheat at the surface, making biphilic surfaces an advantageous area for pool boiling. 

In the second subfigure, the bubbles interact very quickly with each other, coalescing before completing the individual development and detachment. It is now perceptible that there was a temperature drop at the surface that originated from the coalescence of the bubbles causing a sudden movement on the colder fluid. 

**Figure 9:**
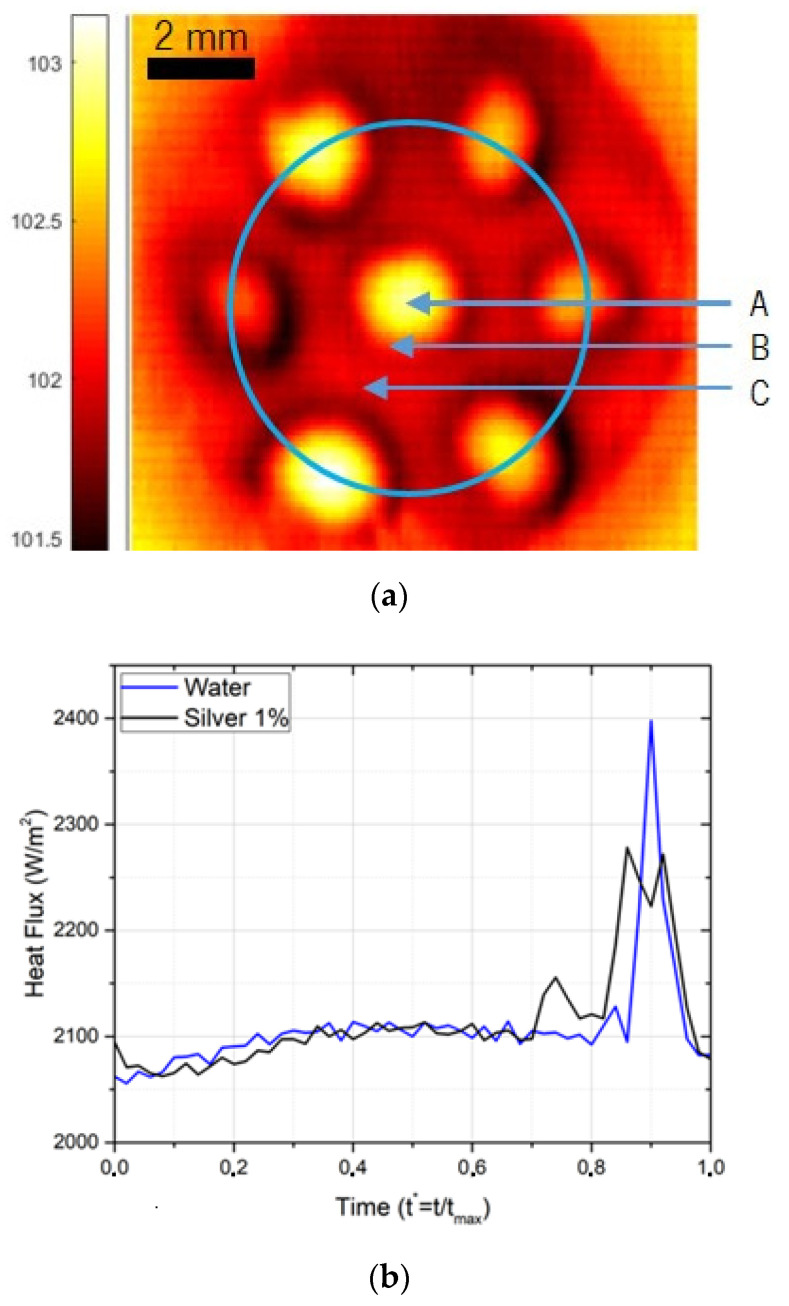
(**a**) Temperature map of the regions of interest for the heat-flux analysis in a biphilic surface with seven superhydrophobic regions. (**b**) Average dissipated heat flux for a septuple superhydrophobic region nucleation. The distance between superhydrophobic regions is *δ* = 1*d*, and the imposed heat flux is 2132 W/m^2^.

For this distance between superhydrophobic regions, which was established following the recommendations of Reference [50], the coalescence actually promotes surface cooling, which is clearly identified in the thermographic images by a temperature drop at the surface, during bubbles detachment and coalescence. The coalescence also helps promote the early detachment of the bubbles, thus increasing frequency and recirculation. This motion of the bubbles promotes fluid motion, thus contributing to the cooling of the surface. To better understand and quantify this trend, three different surface locations were selected for analyzing the temperature evolution during the entire process of nucleation, growth and detachment of a bubble. Moreover, to analyze the dissipated heat flux, a fixed area was selected, to have comparable results. The three chosen locations and selected areas are represented in Figure 9a,b, which depicts the dissipated heat flux obtained for one video with the synchronized bubble-detachment frames.

Firstly, it is important to note that the selected area of interest (as identified in Figure 9a) is considerably larger than the one analyzed for the single bubble case. Thus, the average dissipated heat flux is $q^{\prime \prime }\approx 2100\;W/m^{2}$, which corresponds to a value similar to that imposed to the surface (being lower than that obtained for a single bubble analysis). Moreover, the variation of the dissipated heat flux is different. Hence, on the single superhydrophobic region case, the heat flux reached a maximum value shortly after the bubble detachment, dropping continuously until a new necking phase starts. On the other hand, in Figure 9b, the dissipated flux in the biphilic surface is almost constant, except for a brief instant at t* ≈ 0.9. At this instant, there is a substantial spike in the heat flux, reaching values of $q^{\prime \prime }\approx 2400\;W/m^{2}$. Hence, and as concluded from analyzing the temperature map, the bubble interaction and coalescence on this biphilic pattern has a sizable positive impact on the heat-transfer phenomenon.

**Figure 10:**
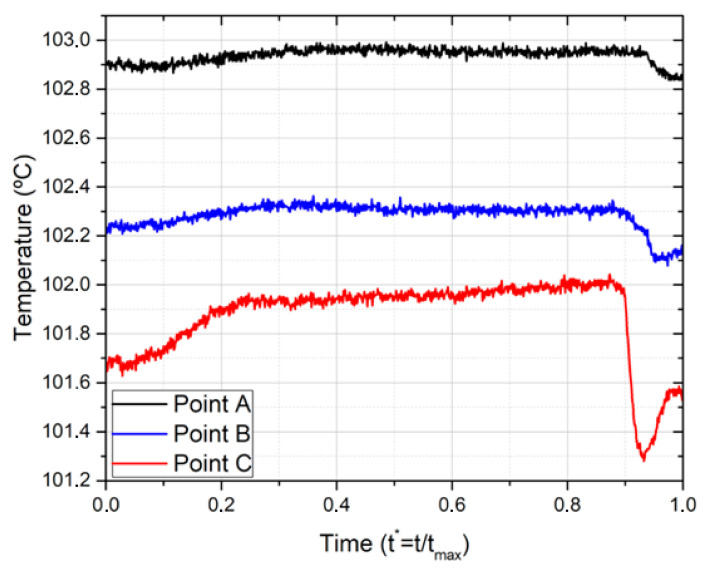
Temperature variation in three points of interest of the biphilic surface. The distance between superhydrophobic regions is δ = 1d, the imposed heat flux is 2132 W/m^2^ and the fluid is Ag 1%.

In Figure 10, the temperature evolution for the selected locations (Figure 9a) is depicted. Points A and B, which respectively represent the center and the boundary, have an almost constant temperature evolution during the nucleation. Only at t* ≈ 0.9 is there a more noticeable change in temperature, which is in line with the coalescence phenomenon described before. Following that, the temperature rises, stabilizing at t^*^ ≈ 0.3. The average temperature is approximately 102.9 °C at point A and 102.3 °C at point B. 

In point C, the results are more significant and depict the influence of bubble interaction. The temperature increases very slowly during almost all the nucleation. However, at t* ≈ 0.9, there is a sudden decrease of temperature, confirming the local cooling due to the coalescence phenomenon. The temperature drops approximately 0.7 °C, and the average temperature is *T* ≈ 101.8°C. 

**Figure 11:**
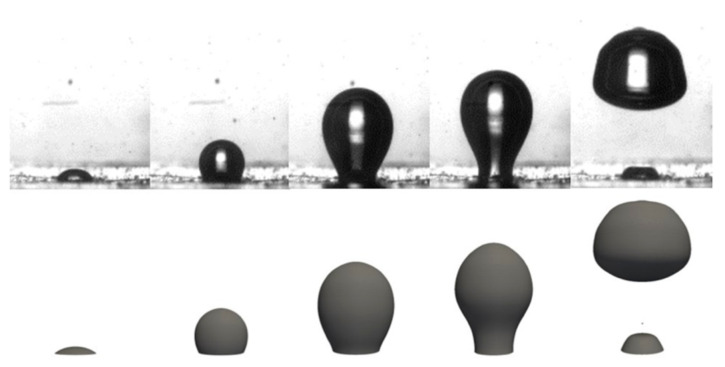
Comparison between experimental and numerical results to validate the model. The phases are water and water vapor, the superhydrophobic region diameter is 1.5 mm and the imposed heat flux is 1290 W/m^2^.

After the analysis of the experimental results concluded, a numerical bubble dynamics model was developed, and its results were compared with the experimental data reported so far. Figure 11 shows this comparison. Since all of the thermophysical properties of water and water vapor are known, the simulation was performed for these two phases. The imposed heat flux is 1290 W/m^2^. 

The pairing of Figure 11 shows a good agreement between the experimental and numerical bubble development. Fivetimeframes are depicted and correspond to the previous described stages of the nucleation, respectively: initial stage, hemisphere formation, vertical elongation, necking and bubble detachment. The numerical case follows these same steps, and, therefore, the bubble shape is similar throughout the nucleation. However, in the fourth pair of images (necking phase), a small deformation of the bubble shape appears for the numerical case. During the experimental bubble growth, some instability is observed, especially at the later stages, when the bubble and, consequently, the vapor mass are of higher values. This seems to be caused by convection currents, as well as the growing surface-tension forces acting on the bubble base. The instability present during bubble growth seems to be slightly enhanced in the numerical model.

The numerical model starts without a defined vapor layer, and an induced cavity was added to start the nucleation. Because of this and since only one bubble detachment is considered, the start of the numerical simulation does not correspond to the start of the nucleation of the experimental setup. Thus, the numerical data were only extracted when the vapor layer totally covered the superhydrophobic region. 

**Figure 12:**
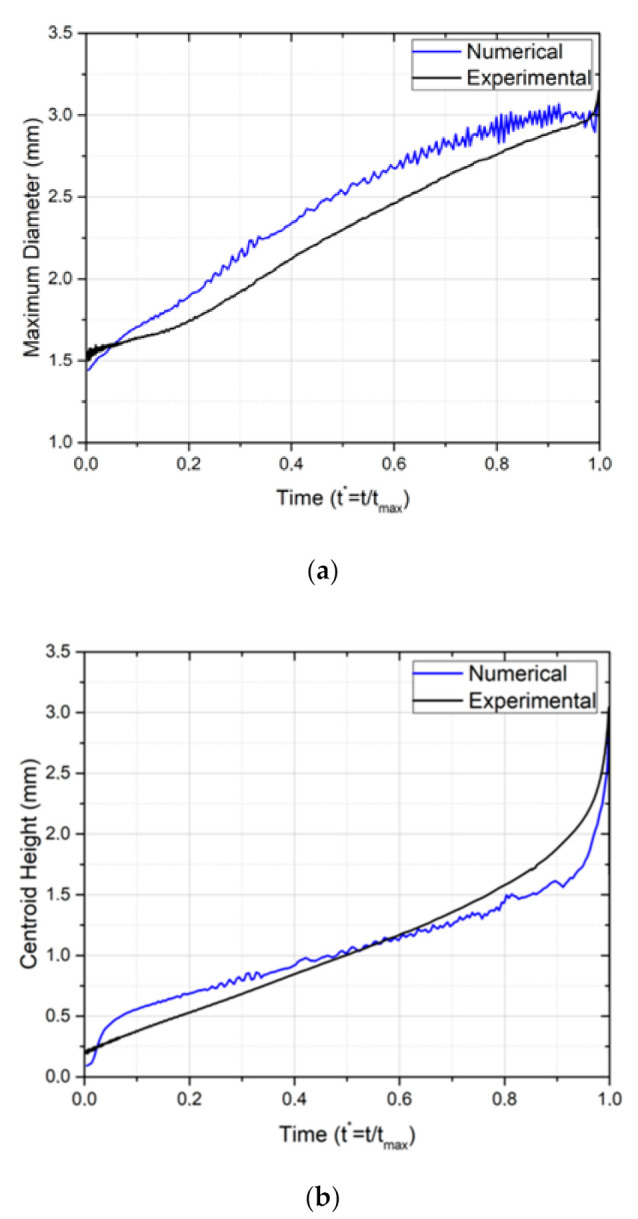
Comparison of bubble dynamic parameters for a superhydrophobic region with a diameter of 1.5 mm, a heat flux of 1290 W/m^2^ and with water as working fluid. (**a**) Temporal evolution of the bubble maximum diameter. (**b**) Temporal evolution of the bubble centroid height.

In Figure 12, it is noticeable that the bubble of the numerical model follows the same stages as the experimental results, for the same conditions, which is in agreement with the analysis of Figure 11.

At the beginning of the nucleation, a fast increase in both diameter and centroid height is noticed for the numerical case—in Figure 12a,b, respectively. The difference may arise from the fact that the numerical simulation considers a bubble rising without a preceding bubble, whilst the experimental results are an average of various nucleations with a continuous vapor layer. The flow of liquid caused by the detaching bubble might slow down the initial stage of the next bubble. Following this, the diameter increases similarly for both cases, with the numerical model presenting a slightly higher bubble diameter, which is not relevant considering the uncertainty associated to the experimental results. As for the centroid height, the bubble of the numerical simulation seems to rise slower than the ones observed experimentally, although following the same tendency. However, and for both parameters, it is clear the instability on the bubble interface. Looking at Figure 12a, we see that the instability increases during the bubble development, and it is at its peak during the necking phase, t* ≈ 0.9.

**Figure 13:**
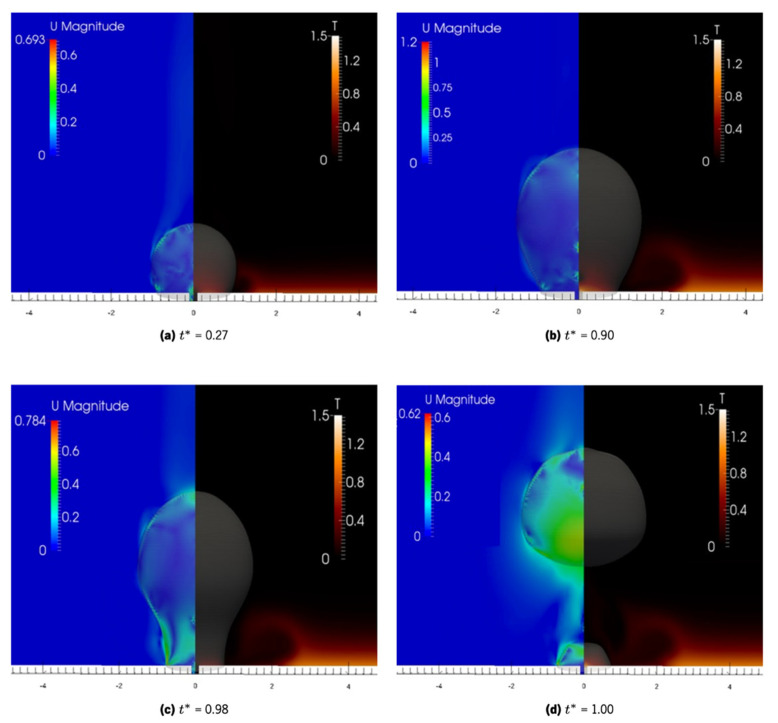
Velocity magnitude and temperature during bubble growth. The imposed heat flux is 1290 W/m^2^, the fluid is water and the superhydrophobic region diameter is 1.5 mm. (**a**) bubble growth at t* = 0.27. (**b**) bubble growth at t* = 0.90. (**c**) bubble necking at t* = 0.98. (**d**) bubble detachment at t* = 1.00.

Figure 13 illustrates the velocity and temperature profiles by using color maps for different timeframes of the nucleation. The velocity magnitude is close to zero in all the domain, which is expected in a pool boiling regime. Only the vapor phase and the interface present some regions where the velocity can be relevant. In Figure 13a,b, the bubble is growing slowly, and therefore the velocity is almost all due to convection currents. In Figure 13c, the strangulation of the base in the necking phase makes the bubble shape change very quickly, and this increase of velocity is beginning to be noticeable in the neck region. Finally, in Figure 13d, the bubble detaches and rises quickly. Therefore, the velocity is higher at the base of the mass of vapor detaching from the surface. 

By analyzing the velocity fields, it is possible to observe a sudden acceleration at the instant of bubble detachment. The temperature on the analyzed region shows the same tendency as the experimental results. However, it is clear that there are two major differences: in the mean temperature value and a local disparity at the center of the bubble. These disparities can be explained, respectively, by the lack of a developed thermal layer in the numerical case and by the artificial nucleation cavity in the numerical case.

## 4. Conclusions

This work presents a fundamental study to infer on the combined use of nanofluids with biphilic surfaces, i.e., hydrophilic surfaces patterned with superhydrophobic regions. The results confirm an effective benefit to use the biphilic pattern to enhance the heat flux dissipation, but only for a well-established distance between superhydrophobic regions, of the order of the characteristic bubble diameter. This was verified in the plot of the dissipated heat flux for a biphilic pattern with seven superhydrophobic regions, δ = 1/d and an imposed heat flux of 2132 w/m^2^. In this case, the dissipated heat flux is almost constant (except in the instant t* ≈ 0.9 when it reaches a peak of 2400 W/m^2^), whilst, in the case of a single superhydrophobic region, the heat flux dissipation reaches the maximum shortly after the detachment phase of the bubble and then will drop in a continuous manner, until the new necking phase. Such distance, δ, allows a controlled bubble coalescence, which promotes fluid convection at the hydrophilic surface between the superhydrophobic regions. This cold fluid motion clearly helps cool down the surface, as observed in the heat-flux peak and surface-temperature maps. This effect was noticeable in the case of employing the Ag 1 wt% nanofluid and an imposed heat flux of 2132 W/m^2^, where the mentioned coalescence contributed to the cooling of the hydrophilic areas, identified by a temperature drop of 0.7 °C, and thus resulted in an average temperature of 101.8 °C (against 102.9 °C on the superhydrophobic spots at the same instant). The effect of the concentration of nanofluids, for the low concentrations employed in this study, plays a minor role, as we noticed a slightly higher heat flux for the nanofluids (Ag 1 wt%), when compared with water, when boiling on the patterned biphilic surface with multiple superhydrophobic regions. This slight effect was also observed when the heat-dissipation decay occurred in the necking stage of the bubble dynamics for nanofluids with different concentrations of the same nanoparticles. For the Au 0.1 wt% nanofluid, it was reported a heat dissipation decay of 350 W/m^2^, whilst for the Au 0.5 wt% concentration nanofluid, it was only of 280 W/m^2^. It is not a significant discrepancy, but nevertheless opens the possibility of further studies in the nanofluids concentration field. 

Finally, the numerical model was validated, and the results for a simulation with water as the working fluid were analyzed. The numerical bubble presented the same phases of nucleation when compared to the experimental results. The analysis of the bubble maximum diameter and the centroid height also allowed us to evaluate the symmetry of the experimental and numerical results, which has allowed us to conclude that the numerical model is valid for this type of simulation. The temperature on the analyzed region shows the same tendency as the experimental results. 

As a closing remark, the authors of this work would like to restate the importance of the dual biphilic patterns–nanofluids cooling systems to industries like the space industry. On the industry of space, the heat accumulation can slow down the functioning of systems, parts, components and chips or even may cause damage. To tackle that malfunctioning (which could be critical in this kind of industry), the smallest and most efficient heating/cooling systems should be developed. As recommended guidelines for future designers and researchers of those systems, the authors of this paper emphasize the enhancement of heat transfer at pool boiling scenarios. This could be done, as confirmed in this work, with the use of small area biphilic pattern surfaces with several superhydrophobic spots evenly separated by a well-established distance correlated with the diameter of the bubble of the cooling fluid. The areas between the mentioned spots should be hydrophilic. Those kinds of pattern surfaces will act more strongly in the heat-dissipation rate than only one superhydrophobic surface. Additionally, nanofluids based upon solutions with higher concentrations of nanoparticles (preferably up to 4%) than the ones used in this work should be used to investigate, with more accuracy, the effect of the concentration on the heat-dissipation decay.

## Figures and Tables

**Table 1:** Thermophysical properties of the working fluids at temperature of 293.15 K at 1 × 10^5^ Pa.

Fluid	Density ρ [kg/m^3^]	Heat Capacity c_p_ [kJ/kg·K]	Surface Tension σ [mN/m]
Pure Water	958.2	4.22	71.6 ± 1.3
Gold 0.1%	959.2	4.21	69.7 ± 3.4
Gold 0.5%	962.9	4.20	74.7 ± 4.3
Silver 0.5%	962.7	4.20	70.9 ± 2.6
Silver 1%	967.3	4.18	72.8 ± 0.8
Alumina 0.05%	958.6	4.21	71.1 ± 3.1
Aliminium 0.05%	958.5	4.21	72.0 ± 7.9

**Table 2:** Mean contact angle results for the superhydrophobic spot for pure water and different nanofluids, at a temperature of 20 °C.

Fluid	Contact Angle Static (Advancing/Receding) θ_s_ (θ_a_/θ_r_) (°)	Hysteresis Δθ (°)
Pure Water	161.8(160.7/159.5) ± 1.8	1.2 ± 2.2
Gold 0.1%	160.6 (162.3/160.1) ± 0.9	2.2 ± 0.7
Gold 0.5%	158.1 (159.1/157.1) ± 5.7	2.0 ±1.2
Silver 0.5%	160.1 (160.6/156.5) ± 0.3	4.1 ± 1.0
Silver 1%	159.7(155.6/153.0) ± 1.6	2.6 ± 0.4
Alumina 0.05%	161.0 (152.9/151.8) ± 2.4	1.1 ± 1.0
Aluminum 0.05%	162.3(160.6/157.4) ± 1.7	3.1 ± 2.2

**Table 3:** Mean surface tension values for pure water and different nanofluids, at a temperature of 20 °C.

Fluid	Surface Tension σ (mN/m)
Pure Water	71.6 ± 1.3
Gold 0.1%	69.7 ± 3.4
Gold 0.5%	74.7 ± 4.3
Silver 0.5%	70.9 ± 2.6
Silver 1%	72.8 ± 0.8
Alumina 0.05%	71.1 ± 3.1
Aluminum 0.05%	72.0 ± 7.9

**Table 4:** Vapor phase properties and surface tension at saturation temperature and atmospheric pressure.

Property	Value
Flux $\nu_{v}\;$(m^2^/s)	2.02 × 10^−5^
Density $\rho_{v}\;$(kg/m^3^)	0.60
Conductivity $k_{v}$ (W/m.K)	0.03
Heat Capacity $C_{p}$ (J/kg.K)	2029
Surface Tension $\sigma_{lv}$ (N/m)	0.06
Heat Transfer $h_{fg\;}$(kJ/kg)	2257

## Supplementary Materials

The following are available online at https://www.mdpi.com/2079-4991/11/1/125/s1.
