# Supplementary material for: Pool Boiling of Nanofluids on Biphilic Surfaces: An Experimental and Numerical Study
Source: Nanomaterials (Basel). 2021 Jan 7;11(1):125. doi: 10.3390/nano11010125 (PMC7826585; doi:10.3390/nano11010125)
Supplement: Supplementary file 1 [file nanomaterials-11-00125-s001.pdf]

nanomaterials

Supporting Information

Pool Boiling of Nanofluids on Biphilic Surfaces: An Experimental and Numerical Study

Eduardo Freitas [1], Pedro Pontes [1], Ricardo Cautela [1], Vaibhav Bahadur [2], João Miranda [3], Ana P. C. Ribeiro [4], Reinaldo R. Souza [5], Jeferson D. Oliveira [6], Jacqueline B. Copetti [7], Rui Lima [3,5], José E. Pereira [1], António L. N. Moreira [1] and Ana S. Moita [1,8,*]

[1] IN+, Center for Innovation, Technology and Policy Research, Instituto Superior Técnico, Universidade de Lisboa, Av. Rovisco Pais, 1049-001 Lisboa, Portugal; a75320@alunos.uminho.pt (E.F.); pedrodanielpontes@outlook.pt (P.P.); ricardo.m.cautela@tecnico.ulisboa.pt (R.C.); sochapereira@tecnico.ulisboa.pt (J.E.P.); aluismoreira@tecnico.ulisboa.pt (A.L.N.M.)
[2] Walker Department of Mechanical Engineering, The University of Texas at Austin, Austin, TX 78712, USA; vb@austin.utexas.edu
[3] CEFT, Faculdade de Engenharia da Universidade do Porto (FEUP), R. Dr. Roberto Frias, 4200-465 Porto, Portugal; jmiranda@fe.up.pt (J.M.); rl@dem.uminho.pt (R.L.)
[4] Centro de Química Estrutural, Instituto Superior Técnico, Universidade de Lisboa. Av. Rovisco Pais, 1049-001 Lisboa, Portugal; apribeiro@tecnico.ulisboa.pt
[5] Metrics, Mechanical Engineering Department, University of Minho, Campus de Azurém, 4800-058 Guimarães, Portugal; reisartre@gmail.com
[6] Center of Technology and Innovation, University Center FSG, Os dezoito do Forte St., 2366, Caxias do Sul P.B. 65020-472, Rio Grande do Sul State, Brazil; jeferson.physics@gmail.com
[7] Mechanical Engineering Graduate Program, LETEF, Laboratory of Thermal and Fluid Dynamic Studies, University of Vale do Rio dos Sinos, Dos Sinos Av., 950, São Leopoldo P.B. 93022-750, Rio Grande do Sul State, Brazil; jaquecopetti@yahoo.com.br
[8] CINAMIL, Department of Exact Sciences and Engineering, Portuguese Military Academy, R. Gomes Freire, 203, 1169-203 Lisboa, Portugal
* Correspondence: anamoita@tecnico.ulisboa.pt

Citation: Freitas, E.; Pontes, P.; Cautela, R.; Bahadur, V.; Miranda, J.; Ana P. C. Ribeiro; Souza, R.R.; Oliveira, J.D.; Copetti, J.B.; Lima, R.; et al. Pool Boiling of Nanofluids on Biphilic Surfaces: An Experimental and Numerical Study. *Nanomaterials* **2021**, *11*, 125. https://doi.org/10.3390/nano11010125

Received: 11 November 2020
Accepted: 31 December 2020
Published: 7 January 2021

Publisher's Note: MDPI stays neutral with regard to jurisdictional claims in published maps and institutional affiliations.

Copyright: © 2021 by the authors. Submitted for possible open access publication under the terms and conditions of the Creative Commons Attribution (CC BY) license (http://creativecommons.org/licenses/by/4.0/).

There are two major sources for the uncertainties related to the diameter measurement. The former is the conversion factor (C_f) from pixel to millimeters and its uncertainty (ΔC_f). This is dependent of the analysis taken with the millimetric paper, and, in order to quantify it, a conservative value of ±5% is considered. The latter is the error associated with the definition of the boundary of the bubble (e_{bd}) in MATLAB, which depends of the threshold value considered. The selected uncertainty value is of ±2 pixel. With this defined, Equation (A1) is used to estimate the error associated with the diameter measurement process:

$$\frac{\Delta d}{d} = \sqrt{\left(\frac{\Delta C_f}{C_f}\right)^2 + \left(\frac{2e_{bd}}{dC_f}\right)^2} \quad \text{(A1)}$$

Table S1 presents the values considered for the relative uncertainties of the parameters used to characterize bubble dynamics. These relative uncertainties were obtained for the same diameter of the superhydrophobic region, for three different fluids and at constant imposed heat flux. These values serve as a reference for the analysis of bubble dynamics using different imposed heat flux values and multiple superhydrophobic regions. The analyzed parameters are the maximum bubble diameter (d), the bubble contact angle (θ), the centroid height (y_c) and the bubble volume (V). The contact angle measurement is another process for which it is necessary to define the uncertainties that can occur. This is mostly influenced by the bubble/surface interface and the tangent lines adjacent to its positioning. The error quantification related to the thermographical analysis is mostly associated with the equipment used. The relative uncertainty values of the different equipment are shown in Table S2.

Table S1. Uncertainty values associated with the parameters of bubble dynamics for different fluids at the same imposed heat flux.

Fluid	e(d) (%)	e(θ) (%)	e(y_c) (%)	e(V) (%)
Water	9.6	21.3	12.7	23.2
Gold 0.5%	7.1	7.1	19.5	22.0
Silver 1%	8.9	22.3	16.1	24.6

Table S2. Uncertainty values associated with the measurement with different types of equipment.

Equipment	Uncertainty
OMEGA DYNE sensor (mbar)	± 1.6
PID * controller (°C)	± 1.0
Type K thermocouples (°C)	± 0.5
Onca MWIR-InSb-320 camera (°C)	± 0.5

* PID—proportional-integral-derivartive.
